# An Analytical Approach for Fast Recovery of the LSI Properties in Magnetic Particle Imaging

**DOI:** 10.1155/2016/6120713

**Published:** 2016-10-26

**Authors:** Hamed Jabbari Asl, Jungwon Yoon

**Affiliations:** ^1^Robots & Intelligent Systems Lab, Gyeongsang National University, Jinju, Republic of Korea; ^2^School of Mechanical & Aerospace Engineering & ReCAPT, Gyeongsang National University, Jinju, Republic of Korea

## Abstract

Linearity and shift invariance (LSI) characteristics of magnetic particle imaging (MPI) are important properties for quantitative medical diagnosis applications. The MPI image equations have been theoretically shown to exhibit LSI; however, in practice, the necessary filtering action removes the first harmonic information, which destroys the LSI characteristics. This lost information can be constant in the *x*-space reconstruction method. Available recovery algorithms, which are based on signal matching of multiple partial field of views (pFOVs), require much processing time and* a priori* information at the start of imaging. In this paper, a fast analytical recovery algorithm is proposed to restore the LSI properties of the *x*-space MPI images, representable as an image of discrete concentrations of magnetic material. The method utilizes the one-dimensional (1D) *x*-space imaging kernel and properties of the image and lost image equations. The approach does not require overlapping of pFOVs, and its complexity depends only on a small-sized system of linear equations; therefore, it can reduce the processing time. Moreover, the algorithm only needs* a priori* information which can be obtained at one imaging process. Considering different particle distributions, several simulations are conducted, and results of 1D and 2D imaging demonstrate the effectiveness of the proposed approach.

## 1. Introduction

Magnetic particle imaging (MPI) is a new method for imaging the spatial distribution of magnetic nanoparticles, as tracers, with high resolution. The method was proposed by Gleich and Weizenecker [1] and exploits the nonlinear magnetization response of the nanoparticles to a time-variable magnetic field and allows for fast image acquisition. MPI has many applications, especially in medical diagnosis such as blood flow visualization for coronary artery diseases, cancer detection [2, 3], stem cell tracking [4], and molecular imaging [5].

MPI uses an oscillating drive field (excitation field) of sufficient amplitude to change the magnetization of the nanoparticles, which induces a voltage signal in the receive coils. To enable spatial encoding of the information, a static magnetic gradient field, also known as the selection field, is utilized in MPI. This field contains a spatial location named a field-free point (FFP) that has zero field magnitude, and only the particles located at the FFP induce the MPI signal in the receive coils [6–8].

Image reconstruction in MPI includes two main approaches [9]. The first approach makes use of the system matrix. This method, also known as frequency space reconstruction, employs a large system matrix and requires its inversion and some postprocessing to deal with poor conditioning. The approach has high computational load and requires the system matrix to be estimated before imaging [10–12]. The second approach is the *x*-space method, which was introduced by Goodwill et al. [13–15]. This approach does not require matrix inversion or precharacterization and hence provides a robust reconstruction algorithm with a potential for real-time imaging.

Linearity and shift invariance (LSI) are important characteristics of most medical diagnostic systems. For example, since X-ray computed tomography (CT) is LSI, the CT images of tissue attenuation coefficient maps provide quantitative lumen diameters for cardiovascular diagnosis [16]. It has been theoretically proven that the MPI is an LSI system [17]. However, in practice, to detect MPI signal, it is necessary to utilize a filter to remove the drive field signal at its fundamental excitation frequency, which is an unavoidable phenomenon in current MPI techniques. This filtering action also removes the induced particle signal at the excitation frequency and hence the complete MPI signal is not available in practice which destroys the LSI properties of the MPI image.

To recover the complete MPI signal to facilitate the use of the fast *x*-space imaging method in qualitative medical diagnosis, an algorithm is presented in [16] and is optimized in [18]. The approaches use the partial field of views (pFOVs), which are primarily generated for enlarging the FOV. It is shown that the lost information can be recovered by matching the two successive overlapped pFOVs, provided the particle concentration at one reference location is known. Although the methods provide satisfactory results, the computational load is reported in [19] to be relatively high, which increases the imaging time and so this cannot be used for ultrafast imaging.

In this paper, the effect of the filtered MPI image is analyzed and it is shown that its value is constant provided that the trajectory of the FFP is generated by a harmonic function, a property that has to be satisfied in both one-dimensional (1D) and multidimensional imaging. Assuming a constant image loss, a fast analytical algorithm is proposed to restore the lost information. The approach can be real time since its complexity depends on solution of a small-sized system of linear equations and does not require overlapping of pFOVs. The method utilizes the derivative of the magnetization curve of the particles and a mathematical model of the *x*-space image. It also requires* a priori* information regarding the system, which can be obtained experimentally or estimated theoretically prior to imaging. This is another advantage of the proposed approach in comparison to previous works, which require a boundary condition to be obtained at the start of imaging. Several 1D and 2D imaging simulations are presented to demonstrate the effectiveness of the proposed algorithm.

## 2. Methods

The developed recovery algorithm is based on the mathematical model of the MPI signal. In this section, the signal model of the *x*-space method is presented and a model of the term removed from the signal as a result of filtering the fundamental frequency of the excitation signal is derived. Then, a new algorithm is proposed to recover the lost image.

### 2.1. Mathematical Model of a 1D MPI Signal

In this section, the mathematical model of the 1D MPI signal is derived which is used in Section 2.2 to model the *x*-space reconstruction method and to analyze the LSI properties of the *x*-space.

When the excitation field in MPI is periodic, the voltage signal *v*(*t*), induced by the particles' magnetization, is periodic as well, and therefore it can be expanded into a Fourier series as follows:

(1) $$\begin{array}{c} v\left(\;t\;\right)=\overset{\infty }{\underset{k=-\infty }{\sum }}V_{k}e^{ik\omega_{0}t}, \end{array}$$

where *V*_*k*_ are the Fourier coefficients and *ω*_0_ = 2*πf*_0_ with *f*_0_ as the frequency of the excitation field. The coefficients can be written as

(2) $$\begin{array}{c} V_{k}=\frac{1}{T}\overset{T/2}{\underset{-T/2}{\int }}v\left(\;t\;\right)e^{-ik\omega_{0}t}dt, \end{array}$$

where *T* = 1/*f*_0_.

Assuming a 1D distribution of the particles in the *x*-direction and a constant sensitivity *ρ*_*x*_ for the receive coil in this direction, the induced voltage by the time-varying magnetization of the particles can be written as follows [17]:

(3) $$\begin{array}{c} v\left(\;t\;\right)=-\mu_{0}\rho_{x}\int_{object}\frac{\partial M\left(x,t\right)}{\partial t}dx, \end{array}$$

where *μ*_0_ denotes the permeability of free space and *M* is the particle magnetization, which depends on the magnetic field *H*(*x*, *t*). The particle magnetization can be modeled using the Langevin function *ℒ*(·) as follows:

(4) $$\begin{array}{c} M\left(\;H\;\right)=mc\left(\;x\;\right)L\left(\;\kappa H\;\right), \end{array}$$

where *m*  [Am^2^] is the particle magnetic moment, *κ*  [mA^−1^] is a property of the magnetic particle, and *c*(*x*)  [particles/m^3^] is the particle concentration that has to be measured in an MPI image. According to (3), the time derivative of magnetization is of interest and can be written as

(5) $$\begin{array}{c} \frac{\partial M}{\partial t}=m\kappa c\left(\;x\;\right)\frac{\partial L\left(\kappa H\right)}{\partial H}\frac{\partial H}{\partial t}. \end{array}$$

On the other hand, the magnetic field that particles experience in MPI is as follows:

(6) $$\begin{array}{c} H\left(\;x,t\;\right)=H_{s}\left(\;x\;\right)-H_{D}\left(\;t\;\right), \end{array}$$

where *H*_*s*_ is the selection field and *H*_*D*_ is the excitation or drive field. Therefore, using (6), (5) can be written as

(7) $$\begin{array}{c} \frac{\partial M}{\partial t}=m\kappa c\left(\;x\;\right)\dot{L}\left(\;\kappa H\;\right)\frac{\partial H_{D}}{\partial t}, \end{array}$$

where $\dot{ℒ}\left(\kappa H\right)=\partial ℒ\left(\kappa H\right)/\partial H$. Now, using (3) and (7), (2) can be written as follows:

(8) Vk=−μ0ρxmκT·∫−T/2T/2∫FOVcxL˙κH∂HD∂tdx e−ikω0tdtVk=−μ0ρxmκT·∫FOVcx∫−T/2T/2L˙κH∂HD∂te−ikω0tdt dx.

By defining the function

(9) $$\begin{array}{c} S_{k}\left(\;x\;\right)≜\frac{-\mu_{0}\rho_{x}m\kappa }{T}\overset{T/2}{\underset{-T/2}{\int }}\dot{L}\left(\;\kappa H\;\right)\frac{\partial H_{D}}{\partial t}e^{-ik\omega_{0}t}dt, \end{array}$$

(8) can be written as

(10) $$\begin{array}{c} V_{k}=\int_{FOV}S_{k}\left(\;x\;\right)c\left(\;x\;\right)dx. \end{array}$$

*S*_*k*_(*x*) are the so-called* system function*, which in fact are the Fourier coefficients of an induced voltage by a point-like distribution of particles at position *x*.

**Remark 1 .**

The region of integration in (3) is “object” if the particles are ideal (i.e., have a step-like magnetization cure). When the particle magnetization is modeled by the Langevin function, the field of view is slightly different from the object to be imaged. Therefore, in the remaining equations, the region of integration is replaced by “FOV.”

For a homogeneous harmonic drive field with cosine waveform as

(11) $$\begin{array}{c} H_{D}\left(\;t\;\right)=A\cos\omega_{0}t \end{array}$$

and a linear selection field *H*_*s*_(*x*) = *G*_*x*_ *x*, where *G*_*x*_ is the gradient of the selection field in the *x*-direction, it has been shown in [20] that *S*_*k*_(*x*) can be written by a convolution as

(12) Skx=−2μ0ρxmκTiL˙κGxx∗Uk−1GxxA1−GxxA2,

where

(13) $$\begin{array}{c} U_{k}\left(\;x\;\right)=\frac{\sin\left(\left(k+1\right)\arccos\left(x\right)\right)}{\sin\left(\arccos\left(x\right)\right)} \end{array}$$

is the Chebyshev polynomial of order *k*. Now, substituting (12) in (10), one has

(14) Vk=−2μ0ρxmκTi∫FOVL˙κGxx∗Uk−1GxxA1−GxxA2cxdxVk=12∫FOVL˙κGxx∗Skidealxcxdx,

where *S*_*k*_^ideal^ represent the system function for the ideal particles, which are defined as

(15) $$\begin{array}{c} S_{k}^{ideal}\left(\;x\;\right)=\frac{-4\mu_{0}\rho_{x}m\kappa }{T}iU_{k-1}\left(\;\frac{G_{x}x}{A}\;\right)\sqrt{1-{\left(\frac{G_{x}x}{A}\right)}^{2}}. \end{array}$$

Now, one can rewrite (14) as follows:

(16) Vk=12∫FOV∫−A/GxA/GxL˙κGxx−x~Skidealx~dx~ ·cxdxVk=12∫−A/GxA/GxSkidealx~·∫FOVL˙κGxx~−xcxdx dx~Vk=12∫−A/GxA/GxSkidealxc^xdx,

where

(17) $$\begin{array}{c} \hat{c}\left(\;x\;\right)≜\dot{L}\left(\;\kappa G_{x}x\;\right)*c\left(\;x\;\right). \end{array}$$

From (15) and (16), one can conclude that *V*_*k*_ correspond to coefficients of a Chebyshev series (see, e.g., [21]), and therefore $\hat{c}\left(x\right)$ can be written based on the Chebyshev series as follows:

(18) $$\begin{array}{c} \hat{c}\left(\;x\;\right)=\frac{G_{x}T}{\pi A\mu_{0}\rho_{x}m\kappa }i\overset{\infty }{\underset{k=1}{\sum }}V_{k}U_{k-1}\left(\;\frac{G_{x}x}{A}\;\right). \end{array}$$

This equation is used in the following subsection to describe the 1D *x*-space reconstruction method.

### 2.2. *x*-Space Imaging and LSI Characteristics

In the *x*-space reconstruction method, the image is defined to be $\hat{c}\left(x\left(t\right)\right)$ at position *x*(*t*) = *x*_FFP_(*t*), where *x*_FFP_(*t*) denotes the position of the FFP.  $\hat{c}\left(x_{FFP}\left(t\right)\right)$ can be measured from the induced voltage *v*(*t*) through the following relation [13]:

(19) c^xFFPt=vtμ0ρxmκGxx˙FFPtt=1/ω0arccosGxxFFP/A,

where ${\dot{x}}_{FFP}\left(t\right)$ is the time derivative of *x*_FFP_(*t*), that is, the velocity of the FFP. The formulation of $\hat{c}\left(x\right)$ by a convolution in (17) demonstrates that the *x*-space MPI image is a linear and shift-invariant image, properties which are characteristic of most clinical imaging systems, for example, ultrasound, CT, and magnetic resonance imaging (MRI). However, to acquire an MPI image while retaining its LSI properties, one needs the measurement of the true value of *v*(*t*), which is not available in practice. In fact, a band-stop filter is used in practice to segregate *v*(*t*) from the signal induced by the drive field (11) in the receive coil. The filter suppresses the first harmonic of the signal at the frequency of the drive field and leaves the signal at higher harmonics.

According to (13) and (18), the filtered first harmonic can be written as [16]

(20) $$\begin{array}{c} \hat{c}{\left(\;x\;\right)}_{lost}=\frac{G_{x}T}{\pi A\mu_{0}\rho_{x}m\kappa }iV_{1} \end{array}$$

which is a constant value in the image. It should be noted that since *V*_1_ is the Fourier coefficient of an odd real function (as a result of the sinusoidal drive field, *v*(*t*) is an odd function), its value is imaginary and therefore, according to (20), the lost image is a real constant value. Using (15) and (16), one can write (20) as follows:

(21) $$\begin{array}{c} \hat{c}{\left(\;x\;\right)}_{lost}=\frac{2G_{x}T}{\pi A}\overset{A/G_{x}}{\underset{-A/G_{x}}{\int }}\hat{c}\left(\;x\;\right)\sqrt{1-{\left(\frac{G_{x}x}{A}\right)}^{2}}dx \end{array}$$

which shows that the lost value varies when $\hat{c}\left(x\right)$ is shifted due to the velocity term $\sqrt{1-{\left(G_{x}x/A\right)}^{2}}$, and hence its envelope at different positions resembles the sinusoidal excitation pattern (note that $\sin\left(\arccos x\right)=\sqrt{1-x^{2}}$). This means that the lost value is maximal at the center of the FOV and minimal at its edges. In the next subsections, the effect of this constant loss is analyzed and a compensation algorithm is proposed.

### 2.3. LSI Analysis

According to the definition, the MPI image is linear when the image intensity is linearly proportional to the concentration of the particles and is shift-invariant when the image intensity is independent of the location of the particles. Considering the expression of the lost image in (21) and the fact that the image loss depends on the location of the particles, it can be verified that the lost information destroys the LSI properties of the MPI image [16].

**Figure 1:**
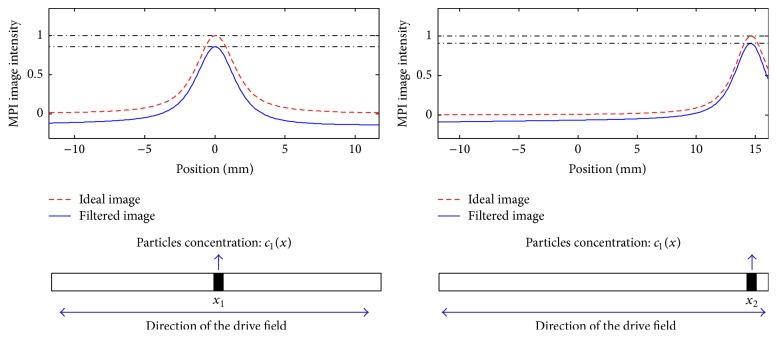
Effect of the filtering on an *x*-space image of particles located at different positions in the FOV.

To visualize the abovementioned phenomenon, consider Figure 1 which shows the *x*-space MPI images of a set of magnetic nanoparticles located at different positions of the FOV with concentration *c*_1_(*x*). As demonstrated, there is a difference in the maximum value of the acquired image intensity for the same particles at different locations. Also, it is clear from this figure that the lost constant value is larger when the particles are located at the center of the FOV, since the velocity of the FFP is maximum at the center.

### 2.4. Recovery Algorithm

Based on the results in the previous section, it can be verified that an *x*-space MPI without first harmonic information does not exhibit LSI and, to provide an image for quantitative medical diagnosis, the lost constant image should be recovered. An algorithm is presented in [16, 18], which can recover the lost image by dividing the FOV mechanically or electronically, into several pFOVs having sufficient overlap to reliably match the successive pFOVs. This overlapping requirement increases the imaging time to cover the entire object. The algorithm also requires an image of a location having no particle, for example, a location outside the patient. Although the method fully recovers the lost image, its computational load may be prohibitive for fast and real-time applications.

**Figure 2:**
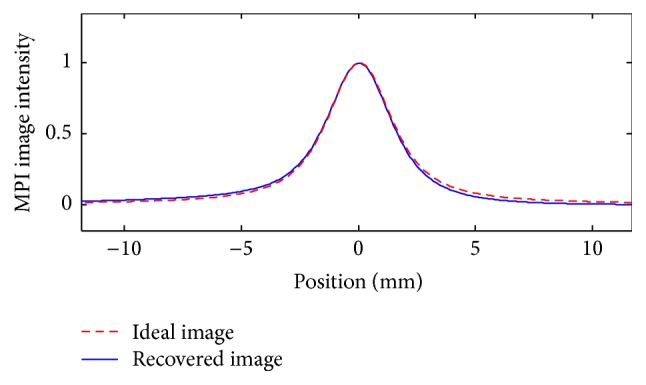
Recovery of the lost constant image, for the example of Figure 1, finding the global minimum of the image.

If the FOV includes a zero-particle area (the reference location for the previous algorithms), finding the lost image is a straightforward task. For example, in MPI images of Figure 1, FOVs have at least one edge at which no particle is located. Therefore, in these cases, finding the global minimum of the image is enough to completely recover the lost image (see Figure 2 for an example). This method, however, fails to completely recover the lost image in general. For example, for the sets of particles shown in Figure 8, only a partial image loss can be compensated by using this method.

The proposed method in this paper is a model-based compensation algorithm, which can be applied in general cases. The approach does not require overlapping of successive FOVs to cover the object, and accordingly it can reduce the imaging time. The method utilizes the model of the point spread function (PSF) of 1D *x*-space image (denoted by $\dot{ℒ}\left(\cdot \right)$), which, as noted in [14], can be obtained theoretically with precise matching with experiment. The algorithm also assumes that the particle distribution can be described as the sum of discrete point-like particles with known locations in the FOV. This is achievable through deconvolution of the original image and looking at the local maxima of the signal to determine the location of particles (a common approach to find the local maxima is to find the first-order difference information of the signal; whenever a smooth signal is available, it is easy to use the available basic algorithms to find the particles, which are very robust; in the simulation results, the “findpeaks” command of MATLAB is used to find the particles and their positions). A deconvolution method is studied for MPI signal in [15]. The reported experimental results of MPI signal show an acceptable noise level to roughly deconvolve the signal for the proposed algorithm [13, 22]. In addition, the approach requires* a priori* information, which can be obtained theoretically or from a reference image, having the same property as used in [16] for initialization. However, unlike the previous studies, this information can be obtained before imaging.

Based on the above explanation, the following assumptions, besides the general assumptions of the *x*-space reconstruction [13], turn out to be crucial within the development of the recovery algorithm.

**Assumption 2 .**

A theoretical model of PSF is available which matches the experimental one.

**Assumption 3 .**

The noise level of the signal is attenuated enough before applying the recovery algorithm.

**Assumption 4 .**

A robust deconvolution method is applied to roughly represent the MPI signal as the sum of signals of discrete particles with definite positions.

In the following, first, an approach is developed for the case in which the particles in the FOV are not in close proximity; that is, the tails of the image for one set do not affect the image of the other sets. Then, the approach is modified for the more general case.

According to (17), the image for the set of point-like particles can be written as

(22) $$\begin{array}{c} {\hat{c}}_{i}\left(\;x_{i}\;\right)=\dot{L}\left(\;\kappa G_{x}x_{i}\;\right)c_{i}, \end{array}$$

where the subscript *i* denotes the *i*th particle. Also, it is obvious from (21) that the image loss for this set of particles (voxel) is proportional to the particle concentration and an integral term depending on $\dot{ℒ}$ and the velocity of the FFP. For point-like particles, the derivative of the Langevin function is nonzero only in a small region, and therefore the velocity term can be assumed to be constant in this region (however, for a case in which the PSF is not very narrow, this assumption is valid up to a certain accuracy). As a consequence, the lost image for the set of particles with concentration *c*_*i*_ located at *x*_*i*_ can be approximated as

(23) $$\begin{array}{c} {\hat{c}}_{i}{\left(\;x_{i}\;\right)}_{lost}\approx k^{\prime }c_{i}\alpha_{i},\;\text{with }\alpha_{i}≜\sqrt{1-{\left(\frac{G_{x}x_{i}}{A}\right)}^{2}}, \end{array}$$

where *k*′ includes the constant terms in (21) and also the integral of the derivative of the Langevin function (which is the PSF of the image). Equation (23) indicates that the image loss for a set of particles is linearly proportional to the velocity of the FFP at the position of the particles, and hence its envelope, throughout the FOV, resembles a sinusoidal pattern.

Using (22), one can write (23) as in the following:

(24) $$\begin{array}{c} {\hat{c}}_{i}{\left(\;x_{i}\;\right)}_{lost}\approx k{\hat{c}}_{i}\left(\;x_{i}\;\right)\alpha_{i}, \end{array}$$

where $k≜k^{\prime }/\dot{ℒ}\left(\kappa G_{x}x_{i}\right)$ is a constant value for all positions in the FOV (the value of PSF, $\dot{ℒ}$, is maximum and constant at *x*_*i*_). This value, which is required in the subsequent development, can be identified theoretically or experimentally through the imaging of a simple set of particles, whose image loss can be easily obtained by finding the global minimum of the signal. In other words, a boundary condition as satisfied in Figure 2, similar to the previous pFOV methods [16], is enough to experimentally measure this value. It should also be noted that, unlike the pFOV methods, this measurement is only required to be done once.

Based on the above result, assuming that the *x*-space imaging conditions are satisfied, the image loss for the particles with concentration *c*_*j*_ located at position *x*_*j*_ can be related to the image loss of the particles with concentration *c*_*i*_ at position *x*_*i*_, as follows:

(25) $$\begin{array}{c} {\hat{c}}_{j}{\left(\;x_{j}\;\right)}_{lost}\approx \frac{{\hat{c}}_{j}\left(x_{j}\right)\alpha_{j}}{{\hat{c}}_{i}\left(x_{i}\right)\alpha_{i}}{\hat{c}}_{i}{\left(\;x_{i}\;\right)}_{lost}. \end{array}$$

Since image $\hat{c}\left(x\right)$ is linear, it can be considered as the sum of the images of the point-like particles distributed throughout the imaging axis [20]. The lost image $\hat{c}{\left(x\right)}_{lost}$ associated with $\hat{c}\left(x\right)$, therefore, can also be considered as the sum of the image losses associated with each set of particles. Then, assuming that $\hat{c}\left(x\right)$ is the image of the *n* sets of particles located in the FOV, it can be written as

(26) $$\begin{array}{c} \hat{c}\left(\;x\;\right)=\overset{n}{\underset{i=1}{\sum }}{\hat{c}}_{i}\left(\;x_{i}\;\right). \end{array}$$

Also, for each individual set, one has

(27) $$\begin{array}{c} {\hat{c}}_{i}{\left(\;x_{i}\;\right)}_{read}={\hat{c}}_{i}\left(\;x_{i}\;\right)-\hat{c}{\left(\;x\;\right)}_{lost}\approx \frac{{\hat{c}}_{i}{\left(x_{i}\right)}_{lost}}{k\alpha_{i}}-\hat{c}{\left(\;x\;\right)}_{lost}, \end{array}$$

where ${\hat{c}}_{i}{\left(x_{i}\right)}_{read}$ is the measured value for the particles noted by index *i* at position *x*_*i*_. Therefore, it can be verified that, for two particle sets of *i* and *j*, the subtraction of measured values, defined by *β*_*ij*_, is equal to the subtraction of the real image values at *x*_*i*_ and *x*_*j*_, respectively; that is,

(28) $$\begin{array}{c} \beta_{ij}≜{\hat{c}}_{i}{\left(\;x_{i}\;\right)}_{read}-{\hat{c}}_{j}{\left(\;x_{j}\;\right)}_{read}={\hat{c}}_{i}\left(\;x_{i}\;\right)-{\hat{c}}_{j}\left(\;x_{j}\;\right), \end{array}$$

which can be measured from the filtered image, based on the assumption that the particles can be identified in the FOV. Now, using (24) and (25), one has

(29) c^jxjlostc^ixi−βijc^ixiαjαic^ixilost≈αjαic^ixilost−kαjβij.

Therefore, considering an initial particle set, noted here by index 1, the total image loss can be written based on ${\hat{c}}_{1}{\left(x_{1}\right)}_{lost}$ as follows:

(30) $$\begin{array}{c} \hat{c}{\left(\;x\;\right)}_{lost}\approx {\hat{c}}_{1}{\left(\;x_{1}\;\right)}_{lost}\left(\;1+\frac{1}{\alpha_{1}}\overset{n}{\underset{i=2}{\sum }}\alpha_{i}\;\right)-k\overset{n}{\underset{i=2}{\sum }}\alpha_{i}\beta_{1i}. \end{array}$$

Now, substituting (27) in (30), the image loss can be measured based on available information as follows:

(31) $$\begin{array}{c} \hat{c}{\left(\;x\;\right)}_{lost}\approx \frac{\gamma {\hat{c}}_{1}{\left(x_{1}\right)}_{read}}{1-\gamma }-\frac{k}{1-\gamma }\overset{n}{\underset{i=2}{\sum }}\alpha_{i}\beta_{1i} \end{array}$$

with

(32) $$\begin{array}{c} \gamma ≜k\overset{n}{\underset{i=1}{\sum }}\alpha_{i}. \end{array}$$

Equation (31) can be applied for cases in which the discrete particles are sufficiently distant from each other such that the tails of the image for one set do not affect the images of other particles. However, when the particles are close to each other, the algorithm should be modified. This issue is discussed below for the case of two particles; however, it can be easily extended to cases involving multiple particles.

Consider the particles *i* and *j* to be very close to each other; therefore, for these particles, (27) will be as follows:

(33) c^ixiread=c^ixi+c^jxi−c^xlost,c^jxjread=c^jxj+c^ixj−c^xlost,

where ${\hat{c}}_{i}\left(x_{j}\right)$ denotes the effect of the image of the particles at position *x*_*i*_ on the image of the particles at *x*_*j*_. Therefore, to find the lost image, the values of ${\hat{c}}_{i}\left(x_{j}\right)$ and ${\hat{c}}_{j}\left(x_{i}\right)$ should be identified. Knowing the PSF, the relations for these values are given by

(34) c^ixj=c^ixiL˙κGxxi−xj,c^jxi=c^jxjL˙κGxxj−xi.

Substituting for ${\hat{c}}_{i}\left(x_{i}\right)$ and ${\hat{c}}_{j}\left(x_{j}\right)$ from (33), (34) can be written as

(35) c^ixj=c^ixiread−c^jxi+c^xlost·L˙κGxxi−xj,c^jxi=c^jxjread−c^ixj+c^xlost·L˙κGxxj−xi,

which is a system of linear equations for the variables ${\hat{c}}_{i}\left(x_{j}\right)$ and ${\hat{c}}_{j}\left(x_{i}\right)$. The solution of this system gives the values of interaction terms which are a function of $\hat{c}(x{)}_{lost}$. Finally, these terms should be subtracted from the “read” values in (28) and (31), and the total lost image can be obtained from (31). In the case of multiple particles with multiple interactions, a similar system of equations as in (35) can be developed, where the number of equations is equal to the number of interaction terms.

The image loss recovery algorithm is summarized as follows.

*Image Loss Recovery for 1D x-Space MPI*

*Require.* The model of the 1D PSF:

(1) In an initialization scanning, find the image loss for one set of particles by measuring the global minimum of the image signal, and then compute *k* in (24). This step can be omitted when the value of *k* is measured theoretically.
(2) Deconvolve the original image to find the particles and their positions in the FOV through a searching algorithm, and then compute *α*_*i*_ using (23) and ${\hat{c}}_{i}{\left(x_{i}\right)}_{read}$ for each set of particles.
(3) Compute the interaction values of each particle set on the neighbors by solving the system of linear equations, such as (35).
(4) Consider a particle set (indexed 1) as the reference set and measure *β*_1*i*_ for all sets using (28), taking into account the effect of the neighbor particles.
(5) Compute the image loss through (31).

**Remark 5 .**

An important note which should be mentioned is that the algorithm requires knowing as narrow convolution kernel (PSF) model (and its image loss to measure *k*) as possible, which can be used to deconvolve the MPI signal to roughly represent it as the sum of discrete point-like particles. Apparently, when the noise level of the signal allows using a narrower PSF, then the error of approximation in (23) would be less, and the overall performance of the algorithm would be improved.

**Remark 6 .**

From the theoretical point of view, any particle set can be considered as the initial set, indexed by 1 in (31). However, in case that a nonideal filter is implemented in practice, the initial set may affect the accuracy of the algorithm. Selecting this set from those in the center of FOV with a medium concentration can be a proper choice. But, in general, the best choice, which depends on the performance of the filter, can be determined from the results of the first experiments (which can be considered as a calibration procedure).

**Remark 7 .**

Considering the case of a nonideal filter, the same procedure as in Remark 6 can be applied to measure the value of *k*; that is, an appropriate choice is to measure it for the particle at the center of trajectory.

**Remark 8 .**

The approach of [16] uses the average of several scans to find the image loss, and it is guaranteed to be robust with respect to the noise, provided that the image noise has zero mean. Similarly, if this condition is satisfied, the assumption of having smooth signal (introduced in Assumption 3), and accordingly the robustness property, for the proposed approach can be readily achieved by using multiple scans.

### 2.5. Evaluation for Multidimensional MPI

Before studying multidimensional MPI, it is worth mentioning that the reason that the lost image is constant in 1D MPI is that the Chebyshev polynomial of order zero *U*_0_(*x*) is constant and does not depend on the spatial position. On the other hand, the *x*-space image can be written as the Chebyshev series (18) since the system function is expressed by the Chebyshev polynomials. This notation for the system function is the direct effect of a cosine (harmonic) drive field. This means that, for other waveforms of the drive field, the system function cannot be written as the Chebyshev polynomials.

Based on the above discussion, it can be verified that, in general, the image loss in multidimensional MPI is not a dc value. However, one method of maintaining a constant image loss in multidimensional *x*-space is to combine the 1D images of the object to generate a 2D/3D image. This method is experimentally studied in [23], which requires only one pair of drive coils and one receive coil.

Using the above method, the image loss would be constant and recoverable for 1D MPI images, but the signal equation of multidimensional MPI differs from that of 1D MPI. Then, a question arises of whether it is possible to use the proposed recovery algorithm for 2D/3D MPI. This question is investigated below by studying the multidimensional MPI signal.

The generalized 3D MPI signal is given by [23]

(36) $$\begin{array}{c} v\left(\;t\;\right)={\left.\;\rho \left(\;r\;\right)mc\left(\;r\;\right)***h\left(\;r\;\right){\dot{r}}_{FFP}\;\right|}_{r=r_{FFP}\left(t\right)}, \end{array}$$

where **r** = [*x* *y* *z*]^*⊤*^ and **h**(**r**) is a matrix PSF which is convolved in 3D (denoted by *∗∗∗*) with the particle distribution. When the multidimensional image is reconstructed from 1D images, which is by a collinear arrangement of detection coil and drive coil trajectory, then the collinear PSF *h*_||_(**r**) should be considered. Assuming the drive field trajectory is in the *x*-direction, one has [23]

**Figure 3:**
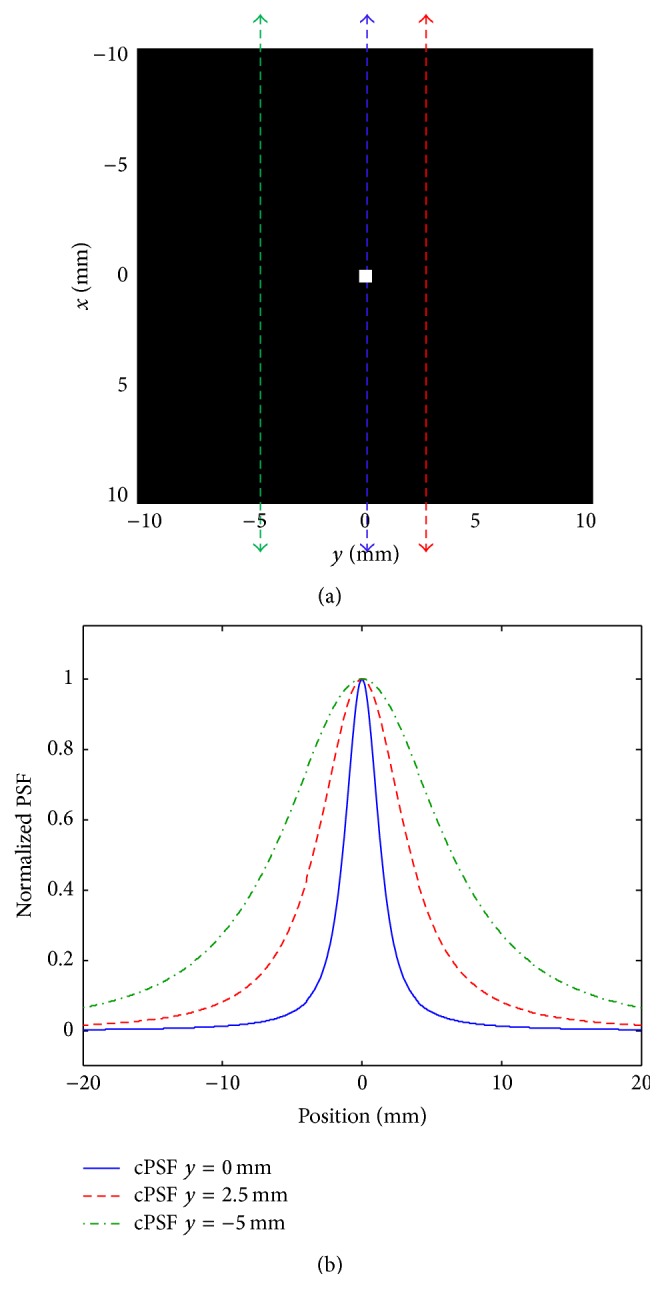
Collinear PSF: (a) a point-like particle and trajectories of the FFP; (b) collinear PSFs at different positions.

(37) h||r=L˙κHrκGx3x2Hr2+LκHrκHr1−Gx3x2Hr2

with $H\left(r\right)=\sqrt{{\left(G_{x}x\right)}^{2}+{\left(G_{y}y\right)}^{2}+{\left(G_{z}z\right)}^{2}}$. Supposing the particles are located at line *y* = *z* = 0, the second term in (37) will disappear when a drive field trajectory is on this line. The remaining PSF is identical to the 1D PSF, which depends on the time derivative of the Langevin function, and is assumed to be known by the proposed algorithm. When the trajectory moves away from this line, the resultant PSF depends not only on the derivative of the Langevin function, but also on the function itself. Therefore, in different spatial positions for the drive field, the PSF would no longer be the same as the one used by the algorithm. However, the collinear PSF is similar to Gaussian curves, which has a narrow shape at line *y* = *z* = 0 and becomes broader with distance from this line.  Figure 3 illustrates a collinear PSF for a point-like sample of particles at different positions of the drive field.

**Figure 4:**
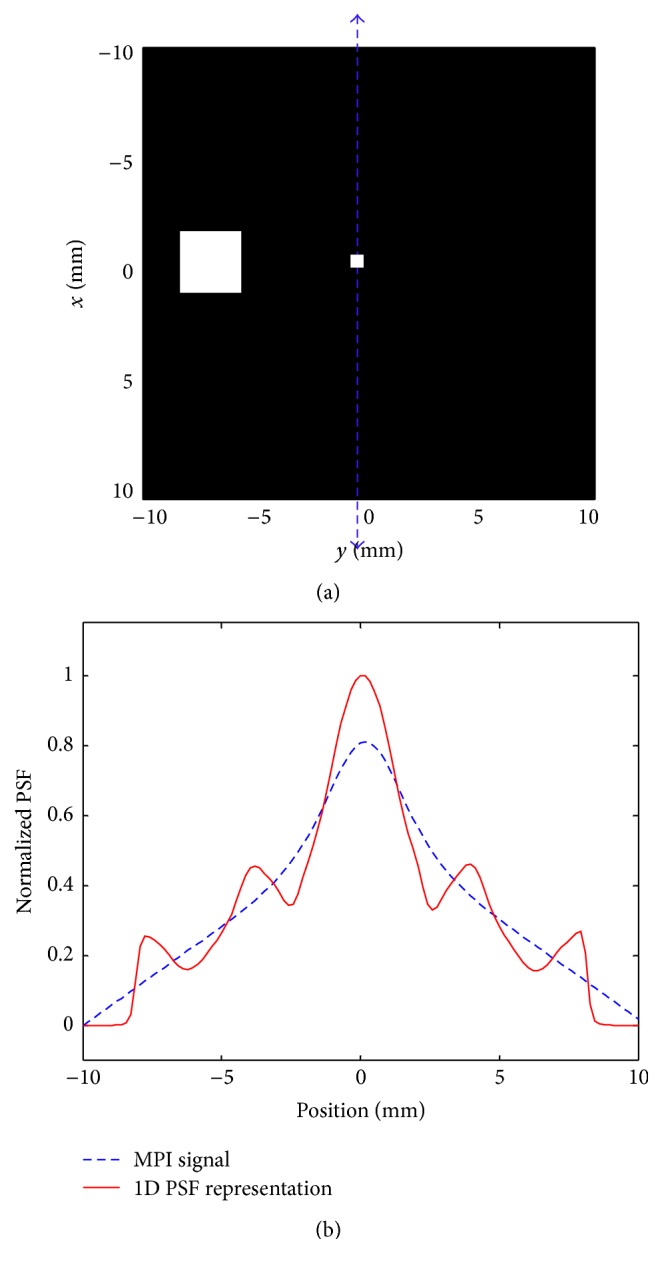
Image representation by 1D PSF: (a) 2D distribution of particles and the position of the drive field; (b) representation of the image signal based on 1D PSF.

It is well investigated in [22] that it is always possible to reconstruct a broad Gaussian-like curve from linear superposition of a narrow one, which is why multicolor imaging is not possible in 1D MPI of Langevin particles with FFP motion being a single line. This property provides the possibility of describing an MPI signal, obtained from a linear FFP trajectory, based on the linear superposition of 1D MPI images resulting from the spatial distribution of the particles along the trajectory line. This distribution can be obtained by deconvolution of the 1D *x*-space PSF from the obtained signal. To clarify this property, an example is illustrated in Figure 4. In this figure, the dashed MPI signal is the output of the band-stop filter, which is induced by the particles at the center of imaging area and the available large set of particles at the border, due to a linear trajectory of the drive field at the center. One should note that although the drive field in Figure 4 does not pass through the large particle set, this set also induces a signal in the receive coil because the FFP is not only a small area for medium-level selection field power, which is generally used in practice. Using a deconvolution algorithm, the solid line in the figure shows a new representation of the signal based on the 1D PSF, which can be interpreted as the superposition of 1D MPI images of several particles with the same PSF. Therefore, this representation property shows that the proposed recovery algorithm is applicable in multidimensional MPI.

**Remark 9 .**

Image of particles with broad PSF, similar to the case where they are close to each other or, in the extreme case, cover the entire FOV, can be expressed as the sum of images of particles with narrow PSF. In fact, the algorithm, regardless of the dimension of particle distribution or imaging, simply tries to find the level of the received signal by expressing the signal as the sum of images of point-like particles (like Figure 4), and then, knowing the effect of a sample set of particles (*k* in (24)), the algorithm estimates the total image loss.

**Remark 10 .**

Nonlinearity of the gradient field or the relaxation effects may cause an asymmetry in the PSF. Considering the point in Remark 9, as long as a model of the PSF is available and the image loss is quasi-constant, the algorithm can still be applied regardless of the asymmetry of the signal. Keep in mind that when the magnetic fields are far from being ideal (ideal magnetic fields: a linear selection field and a homogeneous drive field), the MPI image cannot be formulated as a convolution like (17), and therefore the assumption of constant image loss is not also valid.

## 3. Results and Discussion

**Figure 5:**
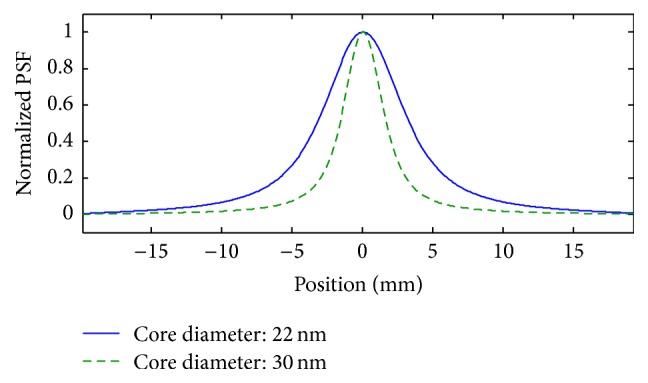
Normalized PSFs for particles with core diameters of 30 nm and 22 nm.

In this section, simulation results are presented to evaluate the effectiveness of the recovery algorithm proposed in Section 2.4. In the following, simulations are first presented for 1D imaging, and then the result for application of the algorithm to a 2D MPI image is illustrated. MATLAB codes are used to simulate both 1D and 2D images. In 1D simulations, point-like particles are considered in the FOV, and since the algorithm is derived based on the shape/model of the PSF, all images are normalized according to the maximum level of concentration; this means the results are reported regardless of the concentration level of the particles. 1D simulations are conducted in three cases for the distribution of particles, and two different particle sizes are analyzed: particles with a core diameter of 30 nm which have a narrow PSF (considered in the first two cases) and particles with a core diameter of 22 nm (considered in the third case). This is to evaluate the error of the approximation used to derive (23). The PSFs for these particles are illustrated in Figure 5.

**Case 1 .**

**Figure 6:**
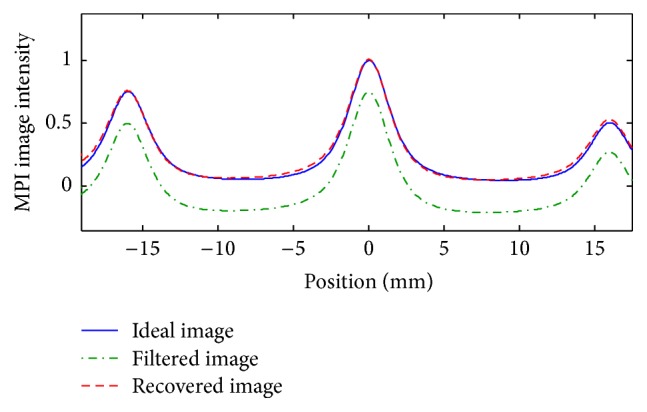
Case 1: filtered 1D *x*-space MPI image and recovered image applying the proposed algorithm for a particle diameter of 30 nm.

As the first study, three sets of particles with different concentrations are considered in the FOV, having no interaction. Since the image of particles does not affect the image of other particles, then the location of particles can be easily determined, and step (3) of image loss recovery for 1D *x*-space MPI is not required. However, since the particles do not have similar concentrations, *β*_*ij*_ must be computed. The result for this case is shown in Figure 6. As the figure demonstrates, in this case, the algorithm can detect the image loss with high accuracy.

**Case 2 .**

**Figure 7:**
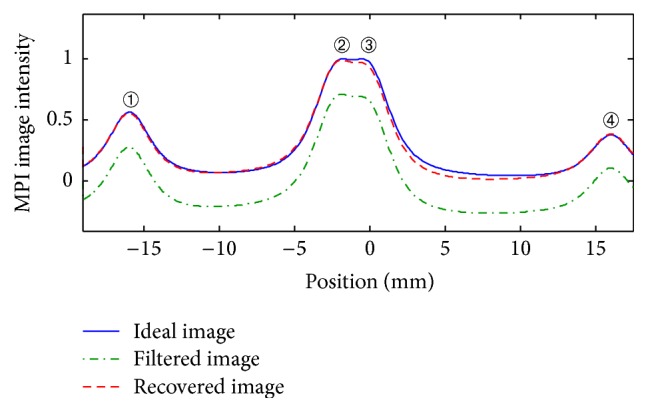
Case 2: filtered 1D *x*-space MPI image and recovered image applying the proposed algorithm for a particle diameter of 30 nm.

In the second case, four sets of particles are considered in the FOV, where particles indexed by “2” and “3” are very close to each other and interact. By deconvolving the image, it is possible to easily determine the position of particles. For this case, it is required to use (35) to cancel the interaction effect. The result for this case is shown in Figure 7, which demonstrates the proper compensation of the lost information. It should be noted that if the particles were closer, then deconvolving could not segregate the particles and they would be considered as one set of particles with a higher concentration.

**Case 3 .**

**Figure 8:**
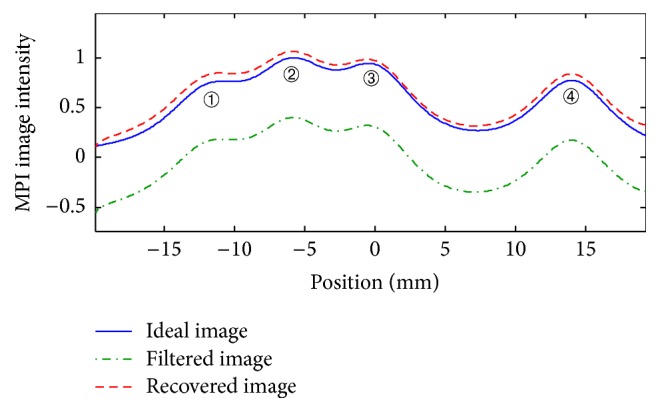
Case 3: filtered 1D *x*-space MPI image and recovered image applying the proposed algorithm for a particle diameter of 22 nm.

In the final case, four sets of particles with a wide PSF are considered in the FOV. In this simulation, the images of particles indexed by “1,” “2,” and “3” undergo interactions with each other. Also, particles “3” and “4” experience interactions. There are a total of eight interactions in the image; therefore, the equivalent model for (35) includes a system of eight linear equations. To simulate the measurement errors, the level of interactions, obtained by (34), is considered to be 90% of its true value. The result of using the algorithm for this case is illustrated in Figure 8, which shows satisfactory recovery of the image, with a small deviation, despite the wide PSF and error in the measurements.

**Figure 9:**
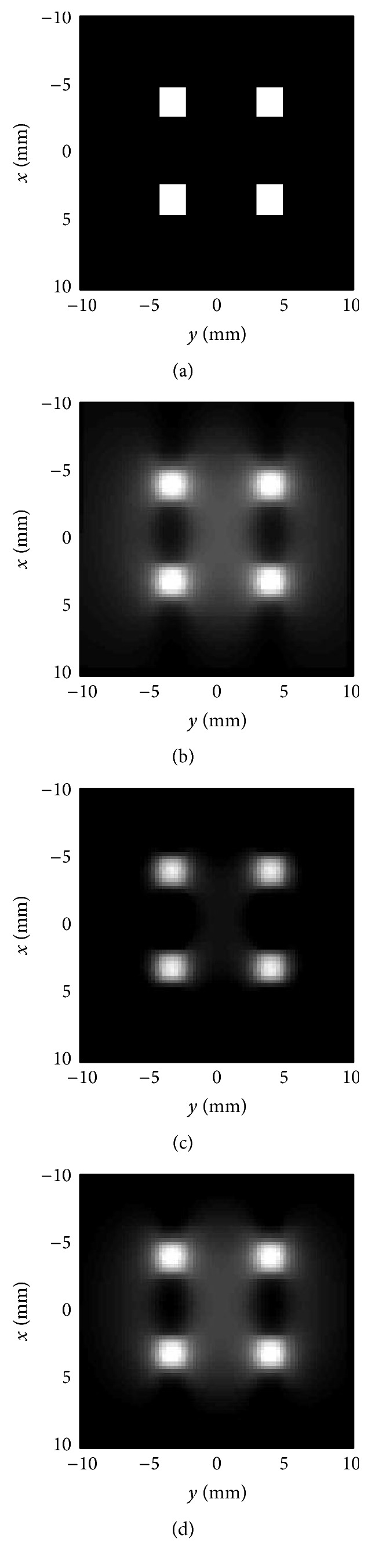
2D image recovery: (a) imaging phantoms, (b) ideal *x*-space image, (c) filtered image, and (d) recovered image.

To simulate the algorithm for 2D imaging, the MPI simulation toolbox developed in [24] is utilized. This MATLAB toolbox simulates the magnetic fields and magnetization of the particles and uses the system function method to reconstruct the image. This program is appropriately modified for the *x*-space imaging and the FFP trajectory is changed to meet the requirements for constant image loss in multidimensional imaging. Measurement error, similar to “Case 3,” is also considered in this simulation.  Figure 9 illustrates the result of applying the recovery algorithm to a 2D image. It is worth mentioning that, in the result, one may expect that Figure 9(c) is better that Figure 9(d), but in fact the concentration of particles shown by Figure 9(d) is more close to the true value. In terms of the image quality, it can be easily improved by combining the images of two orthogonal line-scan drive fields [25].

## 4. Conclusion

This paper analyzes the LSI characteristics of the *x*-space MPI image and proposes an algorithm to recover these characteristics, which are important for quantitative medical diagnosis. Although the theoretical model of the *x*-space image is known to exhibit LSI, it is shown that filtering the fundamental excitation frequency, which is necessary in practice to manifest the particle signal, destroys the LSI properties. The lost image could be constant and hence recoverable when a 1D harmonic drive field is applied in both 1D and multidimensional imaging. To recover the lost information, an algorithm is proposed using *x*-space PSF model of the particles and the properties of the mathematical model of the image. The algorithm tries to find the level of the received signal by expressing it as the sum of images of point-like particles, and then, knowing the image loss of a sample set of particles, the algorithm estimates the total image loss. The initial image loss information can be obtained offline and is enough to be measured for one time. In this regard, the proposed approach has an advantage over the previous methods, where* a priori* information is required to be measured online at the beginning of each test. The complexity of the proposed approach depends on a small-sized system of linear equations, and therefore it has low computational load, which preserves the real-time advantage of *x*-space imaging. This is another advantage of the proposed approach with respect to current available approaches, where overlapping of successive FOVs is required which increases the imaging time to cover the entire object. The results of simulations for 1D and 2D imaging, for both narrow and broad PSFs, demonstrate the effectiveness of the algorithm.

The algorithm is developed based on the assumptions expressed in Assumptions 2–4. Although in simulation study these assumptions are released up to some level and the results are still promising, they seem to be the most challenging parts of implementing the algorithm in practice. In this regard, the future work is devoted to the study, through experiments, of the sensitivity of the algorithm to derive a robustness level with respect to the assumptions. The future work is also devoted to the comparison of the processing times of the proposed algorithm with the previous ones.
